# The Effect of Strict Segregation on *Pseudomonas aeruginosa* in Cystic Fibrosis Patients

**DOI:** 10.1371/journal.pone.0157189

**Published:** 2016-06-09

**Authors:** Rosa van Mansfeld, Angelica de Vrankrijker, Roland Brimicombe, Harry Heijerman, Ferdinand Teding van Berkhout, Cristian Spitoni, Sanne Grave, Cornelis van der Ent, Tom Wolfs, Rob Willems, Marc Bonten

**Affiliations:** 1 Department of Medical Microbiology, UMCU, Utrecht, The Netherlands; 2 Department of Pediatric Pulmonary Diseases, UMCU, Utrecht, The Netherlands; 3 Department of Medical Microbiology, Haga Teaching Hospital, The Hague, The Netherlands; 4 Department of Pulmonology, Haga Teaching Hospital, The Hague, The Netherlands; 5 Department of Pulmonology, UMCU, Utrecht, The Netherlands; 6 Department of Mathematics, Utrecht University, Utrecht, The Netherlands; 7 Department of Pediatric Infectious Diseases, UMCU, Utrecht, The Netherlands; 8 Julius Center for Health Sciences and Primary Care, Utrecht, The Netherlands; University Tuebingen, GERMANY

## Abstract

**Introduction:**

Segregation of patients with cystic fibrosis (CF) was implemented to prevent chronic infection with epidemic *Pseudomonas aeruginosa* strains with presumed detrimental clinical effects, but its effectiveness has not been carefully evaluated.

**Methods:**

The effect of strict segregation on the incidence of *P*. *aeruginosa* infection in CF patients was investigated through longitudinal protocolized follow-up of respiratory tract infection before and after segregation. In two nested cross-sectional studies in 2007 and 2011 the *P*. *aeruginosa* population structure was investigated and clinical parameters were determined in patients with and without infection with the Dutch epidemic *P*. *aeruginosa* clone (ST406).

**Results:**

Of 784 included patients 315 and 382 were at risk for acquiring chronic *P*. *aeruginosa* infection before and after segregation. Acquisition rates were, respectively, 0.14 and 0.05 per 1,000 days at risk (HR: 0.66, 95% CI [0.2548–1.541]; p = 0.28). An exploratory subgroup analysis indicated lower acquisition after segregation in children < 15 years of age (HR: 0.43, 95% CI[0.21–0.95]; p = 0.04). *P*. *aeruginosa* population structure did not change after segregation and ST406 was not associated with lung function decline, death or lung transplantation.

**Conclusions:**

Strict segregation was not associated with a statistically significant lower acquisition of chronic *P*. *aeruginosa* infection and ST406 was not associated with adverse clinical outcome. After segregation there were no new acquisitions of ST406. In an unplanned exploratory analysis chronic acquisition of *P*. *aeruginosa* was lower after implementation of segregation in patients under 15 years of age.

## Introduction

*Pseudomonas aeruginosa* can chronically infect the lungs of patients with cystic fibrosis (CF), contributing to disease progression and death[1]. Based on the presence of highly transmissible *P*. *aeruginosa* strains, so-called epidemic clones, among CF patients[2–7] and presumed detrimental effects of these clones on disease progression[8;9] segregation policies have been implemented for CF patients worldwide. It is thought that segregation prevents acquisition of chronic infections by these CF adapted epidemic *P*. *aeruginosa* clones that are sometimes multi-resistant to antibiotics (i.e. to more than two of the three major classes: beta-lactams, aminoglycosides and quinolones) like the Liverpool Epidemic Strain (LES) and the Australian Epidemic Strain 1 (AES-1) [8;10–12], while strains acquired from the environment can be more easily eradicated[13]. Prevention of these epidemic clones would be beneficial to patients if these clones were indisputably associated with a worse prognosis. This has not been unequivocally demonstrated for so-called “epidemic” clones.

In the Netherlands strict segregation was implemented in 2006, and consisted of strict hygiene rules and the recommendation to avoid contact between all individual CF patients in inpatient and outpatient clinics, as well as outside the hospital setting[14;15]. The decision to implement segregation in the Netherlands was based on findings from abroad and a single Dutch study suggesting transmission of *P*. *aeruginosa* during summer camps[16]. Subsequent cross-sectional investigation of *P*. *aeruginosa* population structure in the Netherlands indicated that 15 percent of the patients were infected with clone ST406, and 5 percent with ST497, which both appeared unrelated to other international CF clones, were not detected in non-CF patients, and ST406 was not associated with unfavorable clinical outcome in a cross-sectional study[17–19].

The effects of strict segregation of CF patients on *P*. *aeruginosa* acquisition and transmission have not been determined systematically. Although reduced prevalence of epidemic clones[20–23], decreased incidence and prevalence of chronic *P*. *aeruginosa* infection[24] and less cross-infection between CF-patients after implementation of cohort segregation[25] have been reported, the possible confounding and interactions between prevalence observations, the dynamics of the population at risk and detailed analyses of pulmonary function in time were not included. In addition, epidemic strains may have different virulence and transmissibility characteristics.

We, therefore, investigated whether strict segregation (1) reduces the acquisition rate of chronic infection with *P*. *aeruginosa*, and, thereby, (2) increases the diversity the population structure of *P*. *aeruginosa* infecting CF patients, and (3) whether infection with the epidemic Dutch ST406 clone is, compared to infection with other types, associated with adverse clinical outcome.

## Materials and Methods

### Ethics Statement

All CF patients in the Netherlands are asked by their clinical doctors to give consent to store their data in a national database for scientific purposes. Almost all patients agree and have given written and informed consent to store their data in this database. This database is administered by the Dutch CF foundation. This database was used to collect patient characteristics and clinical data for the longitudinal lung function analysis and survival analysis.

Culture data was retrospectively collected from the institutions laboratory databases. Cultures were taken routinely by the clinical specialist and *Pseudomonas aeruginosa* isolates from sputum cultures from all CF patients have been stored prospectively since 2005. Retrospectively these *P*. *aeruginosa* isolates have been genotyped. The clinical doctors that treated the patients were not aware of typing results of *P*. *aeruginosa* isolates.

The Institutional Ethical Review Board waived the need for evaluation and approval by the IRB for the retrospective collection of culture data and typing of culture isolates obtained from routine clinical cultures.

Patient culture data was anonymized and de-identified prior to analysis of chronic infection.”

### Patients and Design

Standard of care for CF patients in the Netherlands consist of at least three-monthly visits to the out-patient clinic for physical examination, pulmonary function tests and microbiological cultures of sputum or throat swabs. However, some patients visit less regularly or visit a local hospital instead of the CF clinics for check-ups. Since 2003/2004 all Dutch CF centers use antibiotics for early eradication of *P*. *aeruginosa*, mainly colistin and tobramycin [26], and applied eradication protocols have remained unchanged since 2004. Segregation measures were implemented in the Netherlands during 2006, and included strict segregation of all CF patients in clinical wards and outpatient clinics, in which each patient is physically separated from all other CF patients regardless of *P*. *aeruginosa* colonization status. Outside hospital settings it was recommended not to meet with other CF patients, or to adhere to strict hygiene rules (keep distance, don’t touch, hand hygiene, cough hygiene, don’t share utensils or medication) in case of contact[14;15].

All CF patients that visited the Wilhelmina Children’s Hospital/University Medical Centre Utrecht (UMCU) or the Haga Teaching Hospital (Haga) in The Hague between 2005 and 2011 that had at least 4 years of culture data available (not necessarily consecutive), or were born after 1994, were included in the longitudinal study from the first known culture until the last if this was not performed in 2011 (otherwise included until 31-12-2011). All sputum samples and throat swabs were cultured according to standard diagnostic laboratory protocols of each hospital as described before [17].

Furthermore, two cross-sectional typing studies were performed, using MLST, which included all patients visiting either hospital in 2007 and 2011. Persistence of *P*. *aeruginosa* genotypes was investigated in chronically colonized patients with *P*. *aeruginosa* isolates available for genotyping in both 2007 and 2011. All chronically infected patients who did not undergo lung transplantation and were included in the 2007 cross-sectional study, excluding patients with *Burkholderia species* (n = 10), were included in the nested longitudinal follow-up study. The use of inhaled antibiotics was assessed per year, and expressed as a dichotomous value (yes or no) per year. To account for differences in frequency of pulmonary function measurement, only one Forced Expiratory Volume in one second (FEV_1_) measurement per three months (with the highest value) was included. Lung function values were converted into percent of predicted values for FEV1 based on reference values for either adults[27] or children[28] where appropriate (using only one set of reference values per patient, i.e. the reference set that was appropriate for the majority of measurements). Based on the results of the first cross-sectional study, in 2007, patients were grouped according to *P*. *aeruginosa* genotype in group “ST406” or “other ST” [17].

### Definitions

“Chronic colonization” was defined as the detection of *P*. *aeruginosa* in ≥50% of the respiratory tract cultures per year for two consecutive years with at least three positive cultures. “Intermittent” colonization was defined as *P*. *aeruginosa* isolation from the respiratory tract in <50% of cultures in the previous year, with at least one positive *P*. *aeruginosa* culture in the past[29]. The date of acquisition of chronic *P*. *aeruginosa* colonization was defined as the date of the first *P*. *aeruginosa* isolate within three months before which chronic colonization was established. These definitions are in line with the so-called “Leeds classification”[29]. Chronic *P*. *aeruginosa* acquisition was investigated for the years 2005–2006 (before segregation) and 2007–2011 (after segregation) and expressed as the number of new chronic infections per day among patients at risk (i.e. not chronically infected). Two independent experts and two investigators analyzed anonymized culture data, blinded for patient identifier and calendar dates, to determine acquisition of chronic infection. Discrepancies were reevaluated by all experts and final decisions were reached by consensus. Sequence Types (STs) that were harbored by more than two unrelated CF patients were considered “frequently shared strains”.

### Samples

All sputum samples and throat swabs were cultured according to standard diagnostic laboratory protocols of each hospital as described before [17]. In addition the UMCU also used C-390 disk diffusion (9mm Diatabs^™^, 40mcg) for determination of *P*. *aeruginosa*. One colony of each different colony-morphology (rough/smooth/mucoid and colony size) per sample was randomly picked and stored at -70°C. Isolates of the first stored respiratory tract culture yielding *P*. *aeruginosa* of each patient, during the year 2007 and the year 2011, were genotyped with Multi Locus Sequence Typing (MLST). MLST was used as described before to study genetic relatedness of *P*. *aeruginosa*[2]. Susceptibilities were determined by disk diffusion using EUCAST breakpoints for colistin, tobramycin, ciprofloxacin, ceftazidime, piperacillin + tazobactam in both hospitals[30] and amikacin and meropenem in the UMCU and imipenem in the Haga.

### Multi Locus Sequence Typing (MLST)

MLST was performed according to the protocol by Curran *et al*. [31]. Some adjustments were made, including the use of lysates of the isolates, newly designed primers, adding Q-buffer (Qiagen Benelux B.V., Venlo, the Netherlands) and the use of a touchdown PCR program as described before^E^[17]. PCR products were sequenced on a 3730 DNA Analyzer with the same primers as used for amplification. Sequences were analyzed using Bionumerics 5.1(Applied Maths, St-Martens-Latem, Belgium).

Sequence types (STs) were compared to the *P*. *aeruginosa* Multi Locus Sequence Typing website (http://pubmlst.org/paeruginosa/) developed by Keith Jolley and new alleles and profiles were sent to the curator E. Pinnock[32]. STs that were detected in three or more unrelated patients are named “frequently shared STs”. Relationships between STs were estimated using a Minimum Spanning Tree, based on allelic profiles using the goeBURST-based distances[33], as contained in PHYLOVIZ software[34].

### Statistics

Calculations and analysis were performed using SPSS 20 (SPSS, Chicago, USA) and R (version 2.13.1). Genetic Diversity was calculated by Simpson’s Index of Diversity (ID) as previously described using Bionumerics 5.1 (Applied Maths, St-Martens-Latem, Belgium). A Cox-proportional hazard model with segregation as time-dependent variable with time from birth as time scale was performed to determine the association between segregation and acquisition of chronic colonization with age as covariate. For continuous data the Student’s t-test or Mann-Whitney U-test was used where appropriate. For binominal data the Chi-square test or Fisher’s exact test were used where appropriate. A Cox proportional hazards model was used to study the association between ST406 and time to death or lung transplantation (combined endpoint, whichever occurred first). In case of death following lung transplantation, the date of lung transplantation was designated time of censoring. A (post-hoc) power calculation was conducted with a one sided test a 0.05 level of significance and 0.8 power to calculate the minimum effect (in the HR) one could detect [35]. For longitudinal analyses of lung function, a linear mixed model was used. The model assumed a linear trend in FEV_1_ over time for each patient, and allowed for random patient-specific slope and intercept. Several possible confounding factors were added to the model and tested for significance. A difference in decline of FEV_1_ (slope analysis) between the two groups (ST406 or other clones) was tested by examining the improvement in model fit after adding an interaction term to the model (time*ST group). This interaction term would allow for different slopes over time for the two groups. For the (post-hoc) power calculation R package long power was used to calculate the minimum difference to detect a significant result with a nominal power OD 0.80 and significance 0.05[36]. For analysis of resistance to antibiotic classes between different genotypes the Mann-Whitney U test was used.

## Results

In total, 784 patients were included in the longitudinal follow-up study between January 1^st^ 2005 and December 31^st^ 2011. Of these 117 (15%) were excluded because of insufficient culture results. At the start of the follow-up (1-1-2005) 217 patients were chronically infected with *P*. *aeruginosa*, 39 were infected with *Burkholderia species* or had already received lung transplantation leaving 411 at risk of acquiring chronic *P*. *aeruginosa* infection. Of the 411 patients, 315 were at risk for acquisition of chronic *P*. *aeruginosa* infection between 1-1-2005 and 31-12-2006, while after implementation of segregation (1-1-2007) until end of follow-up (31-12-2011) 382 patients were at risk for acquisition of chronic *P*. *aeruginosa* infection (Table 1 and S1 Table and S1 Fig in online data supplement). There were 11, 17, 13, 5, 5, 3, 2 acquisitions of infection with *P*. *aeruginosa* per year from 2005 till 2011, respectively. The acquisition rate of chronic infection with *P*. *aeruginosa* per year at risk was 0,051 and 0,018 before and during segregation, respectively. The hazard ratio for acquisition of chronic infection during segregation was 0·66 (CI [0·2548–1·541]; p = 0·28), as compared to the risk before segregation. Exploration of the data to explain the wide confidence interval displayed different effects in different age groups with most effect of segregation occurring on younger age groups. Subsequent (post-hoc) subgroup analysis of patients <15 years and >15 years of age resulted in HR of 0·43, (95% CI [0·21–0·95] p = 0·04) and 0·88, (95% CI [0·23–3·34]; p = 0·85), respectively.

**Table 1:** Patient characteristics, microbiological and genotypic characteristics of *P*. *aeruginosa* isolates associated with chronic infection of 411 patients at risk for acquiring chronic infection before (2005–2006) and during (2007–2011) strict segregation of CF patients.

	Patients at risk for chronic infection before segregation (2005–2006) N = 315	Patients at risk for chronic infection during segregation (2007–2011) N = 382
Age at end of follow-up (average and SD)	21·9 (SD 13·2)	18·9 (SD14·0)
Male/female	0·55/0·45	0·54/0·46
Patient days at risk	196,881	551,490
Number of patients with acquisitions of chronic *P*. *aeruginosa* infection	28	28
Rate of acquisitions with *P*. *aeruginosa*/1000 patient days at risk	0·14	0·05
Number of patients with chronic *P*. *aeruginosa* acquisition and isolates genotyped * (%)	20 (71%)	18 (64%)
Number of different sequence types *	18	17
Number of patients with shared sequence types (%)*	12 (60%)	7 (38%)
Number of patients with acquisition of chronic *P*. *aeruginosa* ST 406 infection (%)*	3 (15%)	0 (0%)

* *P*. *aeruginosa* isolates were not available for genotyping from all patients with chronic acquisition. Therefore numbers are based only on all patients with genotyped isolates.

### Population Structure Pseudomonas aeruginosa

The results of the first cross-sectional study (isolates from 2007) have been published[17]. The second identically conducted cross-sectional (point) prevalence study, of isolates obtained in 2011, included 631 patients (97% of all patients visiting either hospital that year) of which respiratory tract cultures were available (391 of 405 in UMCU (97%) and 240 of 244 in Haga (98%)) (Table 2). *P*. *aeruginosa* isolates from 168 patients were available for genotyping both in 2007 and 2011, 96 had isolates only in 2007 and 112 only in 2011. The prevalence of patients infected with *P*. *aeruginosa*, of patients with multiple genotypes and the proportion of patients harboring STs that were shared with more than two unrelated patients were comparable in both study cohorts (Table 2). The prevalence of ST406 was 15% in 2007 and 14% in 2011, but the average age of patients infected with ST406 increased from 19·8 years [range 11·0–30·5] in 2007 to 23·4 years [range 16·7–38·3] in 2011. A Minimum Spanning Tree based on 211 STs representing 599 isolates from Dutch CF patients revealed no specific sub-clustering according to year of isolation (S2 Fig in online data supplement).

**Table 2:** Demographic and microbiological characteristics of cystic fibrosis patients included in cross-sectional studies in 2007 and 2011. (* = p<0.05).

	2007 (n = 551)	2011 (n = 631)
Average age (standard deviation) [range]	23·4 (13·8) [1·0–69·1]	22·6 (14·0) [0–72·2]
Proportion age <18/≥18	0·42/0·58	0·41/0·59
Proportion male/female	0·53/0·47	0·51/0·49
Number of respiratory tract cultures/year, [median, interquartile range]	3 [2–4]	4 [2–6] *
Number of *P*. *aeruginosa* isolates	443	414
Number of patients with intermittent or chronic infection with *P*. *aeruginosa* in the respiratory tract (%)	313 (57%)	326 (52%)
Number of patients with *P*. *aeruginosa* genotyped (% of infected)	265/313 (85%)	280/326 (86%)
Number of different sequence types	143	157
Number of patients with multiple phenotypically different *P*. *aeruginosa* isolates (% of patients with *P*. *aeruginosa* genotyped)	135/265 (51%)	113/280 (40%)
Number of patients infected with multiple *P*. *aeruginosa* sequence types (% of patients with *P*. *aeruginosa* genotyped)	28/265 (11%)	22/280 (8%)
Number of patients with frequently shared *P*. *aeruginosa* sequence types in more than two patients [number of different sequence types]	150 (57%) [24]	140 (50%) [22]
Number of patients infected with *P*. *aeruginosa* ST406 (% of patients with *P*. *aeruginosa* genotyped)	41 (15%)	38 (14%)
Number of patients infected with *P*. *aeruginosa* ST497 (% of patients with *P*. *aeruginosa* genotyped)	14 (5%)	13 (5%)
Simpson Index of diversity [95%CI] of *P*. *aeruginosa* isolates based on MLST	97·3 [96·2–98·4]	97·8 [96·9–98·7]

**Fig 1:**
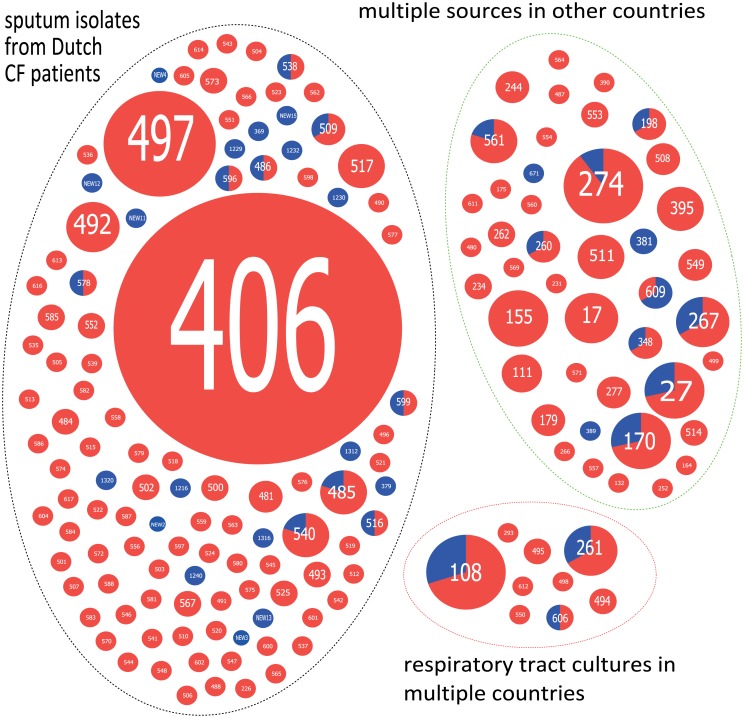
Distribution of *P*. *aeruginosa* genotypes. Distribution of 163 *P*. *aeruginosa* STs detected in 265 Dutch CF patients in 2007 and 49 patients with acquisition after segregation in 2011 among defined source groups. The 41 STs detected in 49 patients with acquired infection during segregation are indicated in blue and STs detected in the cross-sectional study in 2007 are depicted in red. Each circle represents an ST and size of circles represents number of isolates. Dotted circles enclose defined source groups. Assignment of STs to source groups is based on source group definition of STs, contained in the international pseudomonas MLST database (http://pubmlst.org/paeruginosa/), that are identical to the 163 STs identified in Dutch CF patients.

There were 49 patients that acquired *P*. *aeruginosa* during segregation (mostly resulting in intermittent colonization), most frequently with ST108 (n = 3 patients, not related), but not with ST406 and ST497 genotypes, the Dutch prevalent clones (Fig 1). The Simpson index of diversity of these isolates acquired during segregation was 99.2 (98.5–99.9), which is significantly higher than in the whole tested populations. However, there were three acquisitions of chronic infection with ST406 after segregation in patients already chronically infected with non-ST406 *P*. *aeruginosa* in 2007. STs detected in 2007 and 2011 had similar distribution among three defined sources (i.e. sputum isolates from CF patients in the Netherlands, respiratory tract cultures in multiple countries, and other sources from multiple countries) (Fig 1).

### Clinical Effects of ST406

In 2007 219 patients had chronic *P*. *aeruginosa* infection with genotype data available and all were included in the nested longitudinal analysis of clinical outcome, yielding a total follow-up of 693·3 patient years. In 2007 the 40 patients infected with ST406 were, as compared to those infected with other STs, younger and were more likely to have used inhaled antibiotics (Table 3).

**Table 3:** Characteristics of patients infected with ST406 and non-ST 406 *Pseudomonas aeruginosa* genotypes.

	ST 406 (n = 40)	Other clones (n = 169)	p
Age, mean (SD)	18·9 (3·7)	25·0 (13·5)	0·005^a^
Male, n (%)	20 (50)	91 (51)	0·92^b^
Homozygosity dF508, n (%)	30 (75)	98 (61)	0·10^b^
ABPA, n (%)	3 (7·5)	34 (19)	0·08^b^
CFRD, n (%)	9 (23)	40 (22)	0·98^b^
Hospitalizations, median (IQR)	0 (0–1)	0 (0–1)	0·20^c^
FEV1, mean (SD)	67·3 (27·3)	61·9 (23·5)	0·35^a^
BMI z-score, mean (SD)	-0·5 (1·2)	-0·4 (1·1)	0·53^a^
Pulmozyme, n (%)	18 (45)	86 (48)	0·73^b^
Inhaled antibiotics, n (%)	32 (80)	114 (64)	0·048^b^
Age at diagnosis, median (IQR)	0 (0–3)	0 (0–4)	0·25^c^

^a^t-test

^b^Chi^2^

^c^Mann Whitney U

Abbreviations: SD = standard deviation, ABPA = Allergic bronchopulmonaryaspergillosis, CFRD = cystic fibrosis related diabetes, IQR = interquartile range, FEV1 = Forced Expiratory Volume in one second.

**Fig 2:**
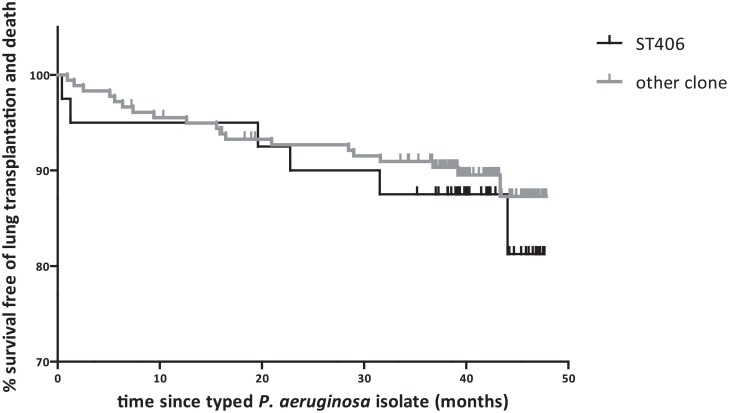
Survival free of lung transplantation or death. Survival free of lung transplantation or death for CF patients with ST406 and CF patients with other *P*. *aeruginosa* clones (total n = 219, lung transplantation n = 11, died n = 14).

Lung function measurements between 2007 and 2010 were available for 201 (92%) patients (n = 1903, mean 9·5 measurements per patient) and the means of FEV_1_ percent of predicted for patients with ST406 and with other STs did not decline and was not significantly different between both groups (S3 Fig in online data supplement). Including age and use of inhaled antibiotics or time on inhaled antibiotics in the linear mixed model did not change interpretation (S2 Table in online data supplement). The hazard ratio (HR) for dying (n = 14) or receiving a transplant (n = 11) was not significantly different for patients with ST406 or with other STs (HR 2·41, 95% CI 0·85–6·88, *p* = 0·10) (Fig 2).

*P*. *aeruginosa* ST406 isolates (n = 51) were resistant to a median of 3 (inter quartile range (IQR) 2–3, maximum 5) of five antipseudomonal antibiotic drug classes (aminoglycoside, betalactam/betalactamase inhibitor combination, antipseudomonal cephalosporin, carbapenem and fluoroquinolone), as compared to 1 (IQR 1–2, maximum 5) for other STs (n = 302) (p<0·0005). Frequently shared STs were more resistant than non-frequently shared STs (n = 181) (median 2, [IQR 1–3]and 1 [IQR 1–2]for non-frequently shared STs (n = 172) (p = 0·003)).

In 158 chronically infected CF patients for which *P*. *aeruginosa* isolates were available for genotyping in both 2007 and 2011, 173 different STs were detected, and 156 STs (90%) were detected in the same patient in 2007 and 2011. Clonal persistence was 89% and 83% for ST406 and other STs, respectively (p = 0·4). Fifteen patients (9%) acquired a new ST, which occurred in addition to persistence of another ST in six patients.

## Discussion

This longitudinal study of seven years failed to demonstrate a statistically significant reduction in acquisition of chronic *P*. *aeruginosa* infection in Dutch CF patients during the first five years after implementation of strict segregation. In two nested cross-sectional studies the population structure of *P*. *aeruginosa* among CF patients remained largely unchanged, characterized by a high genetic diversity of the *P*. *aeruginosa* population among CF patients. *P*. *aeruginosa* ST406, the most prevalent ST in this patient cohort, appeared highly persistent and associated with resistance to more antibiotic classes but infection with this strain was not associated with a detectable decline in pulmonary function or a statistically significant reduced survival free of transplantation.

In crude analysis there was a clear reduction in the incidence of acquisitions of chronic *P*. *aeruginosa* infection after segregation, with a trend towards a statistically significant reduction in Cox regression analysis. Yet, the effects of segregation were different in different age groups, with a more pronounced effect in children. As these cutoff values for age were defined arbitrarily and post-hoc, they should be considered as exploratory. A possible explanation for a larger effect in younger patients is the absence of close contacts between CF patients during CF summer camps, that were no longer organized[16]. Documented lack of adherence to hygiene rules outside the hospital setting by some adolescent and adult patients is a plausible alternative explanation for the lack of effect of segregation in this age group[37]. Furthermore, parents are probably more likely to enforce segregation rules in younger children, and chronic colonization of the sinuses with *P*. *aeruginosa* genotypes acquired before segregation might occurred in the older age groups and include intermittently colonized patients[38–40]. The association of ST406 with a specific age category that was described before[17] is likely a cohort phenomenon, indicating that cross-transmission of ST406 in a certain age-group had occurred as the average age of patients infected with ST406 increased with time.

A marked reduction of cross-transmission of *P*. *aeruginosa* clones would increase the genetic diversity of the *P*. *aeruginosa* population structure. The observed absence of change in Simpson Diversity Index after segregation, corroborates the observed absence of a significant change in acquisition rate. Yet, since already chronically infected patients are known to harbor the same strain for many years, a significant change might not be expected during four years. The diversity of *P*. *aeruginosa* in newly infected patients was significantly higher, however, this group is not directly comparable to the whole tested CF population including intermittent and chronically infected patients. After segregation there were no new acquisitions of ST406 or ST497. This suggests that segregation can prevent spread of epidemic clones, but that segregation does not influence acquisition of strains from the environment. Indeed, the shared strains acquired after segregation mainly belonged to small clusters most likely originating from either common source exposures (e.g. siblings) or the physical environment.

Our data demonstrate that frequently shared strains, especially ST406, are resistant to more antibiotics. Multi-resistance to antibiotics has also been described for the Liverpool Epidemic Strain (LES)[12] and the Australian Epidemic Strain (AES-1)[10]. In our cohort, patients infected with ST406 more frequently received inhaled antibiotics. As clinicians were not aware of *P*. *aeruginosa* genotypes, prescription of inhaled antibiotics cannot be confounded by prior knowledge.

Patients chronically infected with ST406 were younger and had better lung function than older patients with other genotypes. However, the slope of FEV1 over time did not differ between the two groups indicating that the genotype was less important than age for causing a difference in baseline lung function. Yet, the group of patients with chronic ST406 colonization was relatively small and based on post-hoc power calculation a 1.32 difference in slope would have been needed to detect a statistically significant difference.

There are no randomized controlled trials in which the effectiveness of segregation measures for CF patients is evaluated. Yet, many countries have implemented cohort-segregation and in several reports reductions in transmission of epidemic clones, of *P*. *aeruginosa* prevalence and of acquisition of chronic *P*. *aeruginosa* infection at an older age have been reported [20;21;24;25]. As, in addition to cohort-segregation, other interventions were implemented, such as earlier and more stringent antibiotic treatment, more frequent monitoring and screening of newborns for CF, it remains difficult to accurately quantify the effect of segregation from these studies.

Strengths of our study include the longitudinal design, the large patient cohort, comprising half of the Dutch CF population, the completeness of clinical and microbiological data collection and the use of MLST for genotyping. The five-year period of segregation might be considered a study limitation, but the (limited) magnitudes of the effects indicate that much longer follow-up periods would have been needed to reach statistical significance in any of the study endpoints for the total cohort. A changed prevalence of endemic and non-endemic *P*. *aeruginosa* among CF patients, could act as a confounder through a reduced colonization pressure. Yet, segregation does not influence the availability of *P*. *aeruginosa* in other sources than CF patients, and the observed prevalence of *P*. *aeruginosa* in the CF patient population was 57% in 2007 and 52% in 2011, making an important role of colonization pressure highly unlikely. Only post hoc analysis, based on the finding of unequal distribution of effect amongst different age groups, yielded an effect of strict segregation in young CF patients. Segregation did not start on a single day and the pragmatically chosen cut-off point for implementation of segregation on 1-1-2007 is in practice probably an ongoing process of better adherence to the regulations that started to be implemented during 2006. The total number of chronic acquisitions is quite low which might influences the lack of statistical difference before and after segregation. The steep decline from 13 chronic acquisitions to 5 in 2007 and 2008 respectively might indicate ongoing improvement of segregation. Furthermore we have no data on patient behavior and adherence to hygiene rules in their private lives or during hospitalization. Yet, segregation measures have the highest priority amongst hospital staff during hospitalization of CF patients and separate hospital rooms and accommodating segregation in out-patient clinics is rigorously planned. Also we only included CFTR gene defects, but not so-called modifier genes in our analyses. CFTR genotype alone may correlate poorly to lung disease and environmental factors and modifier genes may also be important for disease severity [41]. Acquisition of chronic infection has been associated with heritability in twin studies and with modifier genes like DCTN4, encoding a dynactin protein in large scale sequencing studies[42;43].

Finally, misclassification of infection status may have occurred as only single morphologically different colonies were selected for genotyping and as throat swabs were allowed for detection of lower airway infection with *P*. *aeruginosa*. However, in 80% of those cases in which morphologically different colonies were typed genotypes were identical and > 90% of culture results were from sputum samples.

The conclusion of this study is dual; on the one hand we did not detect new acquisitions with the epidemic ST406 strain (which is a strictly CF related strain) among non-infected patients after segregation, indicating segregation prevents spread of epidemic clones. But the clinical consequences of infection with ST406 seem marginal. On the other hand, only patients under 15 years of age had a significant decrease in chronic acquisition of *P*. *aeruginosa* after implementation of segregation in an unplanned exploratory analysis.

## Supporting Information

S1 Fig Diagram indicating CF patients at risk for chronic colonization with *P*. *aeruginosa* between 2005 and 2011 (i.e. all intermittent and PA negative CF patients). (TIF)

S2 Fig Minimum spanning tree based on allelic profiles of 211 STs representing 599 *P*. *aeruginosa* isolates from Dutch CF patients. Genetic linkage was done using goeBURST distances[33] Each circle represents a sequence type, the size of the circle represents the number of isolates (non-linear). Color of the circle indicates year of origin; blue: 2007 only, green: 2011 only, red: isolated in both 2007 and 2011. Edges connect genetically linked STs.
(PDF)

S3 Fig Mean FEV_1_% of predicted during longitudinal follow-up. Dotted lines represent 95% CI’s. Longitudinal lung function measurements (mean FEV_1_ as percentage of predicted) for patients with ST406 and patients with other clones. There was no significant difference in lung function for patients with or without ST406 (estimate -0·43, 95% CI -7·84–6·98). Adding an interaction term between ST406 and time (slope analysis, not shown in figure) did not significantly improve the model (p = 0·35) indicating no difference in decline.
(PDF)

S1 Table CF patients at risk for chronic colonization with *P*. *aeruginosa* between 2005–2011. PA = *P*. *aeruginosa*, LTX = lung transplantation, * at risk are all patients without chronic PA infection.
(DOCX)

S2 Table Estimates of FEV_1_ percent of predicted based on mixed model analysis. (DOCX)

## References

[1] Folkesson A, Jelsbak L, Yang L, Johansen HK, Ciofu O, Hoiby N, et al Adaptation of *Pseudomonas aeruginosa* to the cystic fibrosis airway: an evolutionary perspective. Nat Rev Microbiol 2012 12;10(12):841–51. 10.1038/nrmicro2907 23147702

[2] Cheng K, Smyth RL, Govan JR, Doherty C, Winstanley C, Denning N, et al Spread of beta-lactam-resistant *Pseudomonas aeruginosa* in a cystic fibrosis clinic. Lancet 1996 9 7;348(9028):639–42. 8782753 10.1016/S0140-6736(96)05169-0

[3] Jones AM, Govan JR, Doherty CJ, Dodd ME, Isalska BJ, Stanbridge TN, et al Spread of a multiresistant strain of *Pseudomonas aeruginosa* in an adult cystic fibrosis clinic. Lancet 2001 8 18;358(9281):557–8. 11520529 10.1016/s0140-6736(01)05714-2

[4] McCallum SJ, Corkill J, Gallagher M, Ledson MJ, Hart CA, Walshaw MJ. Superinfection with a transmissible strain of *Pseudomonas aeruginosa* in adults with cystic fibrosis chronically colonised by *P aeruginosa*. Lancet 2001 8 18;358(9281):558–60. 11520530 10.1016/s0140-6736(01)05715-4

[5] Armstrong D, Bell S, Robinson M, Bye P, Rose B, Harbour C, et al Evidence for spread of a clonal strain of *Pseudomonas aeruginosa* among cystic fibrosis clinics. J Clin Microbiol 2003 5;41(5):2266–7. 12734299 10.1128/JCM.41.5.2266-2267.2003 PMC154738

[6] Fothergill JL, Walshaw MJ, Winstanley C. Transmissible strains of *Pseudomonas aeruginosa* in cystic fibrosis lung infections. Eur Respir J 2012 7;40(1):227–38. 10.1183/09031936.00204411 22323572

[7] Aaron SD, Vandemheen KL, Ramotar K, Giesbrecht-Lewis T, Tullis E, Freitag A, et al Infection with transmissible strains of Pseudomonas aeruginosa and clinical outcomes in adults with cystic fibrosis. JAMA 2010 11 17;304(19):2145–53. 10.1001/jama.2010.1665 21081727

[8] Jones AM, Dodd ME, Doherty CJ, Govan JR, Webb AK. Increased treatment requirements of patients with cystic fibrosis who harbour a highly transmissible strain of *Pseudomonas aeruginosa*. Thorax 2002 11;57(11):924–5. 12403871 10.1136/thorax.57.11.924 PMC1746227

[9] Al-Aloul M, Crawley J, Winstanley C, Hart CA, Ledson MJ, Walshaw MJ. Increased morbidity associated with chronic infection by an epidemic *Pseudomonas aeruginosa* strain in CF patients. Thorax 2004 4;59(4):334–6. 15047956 10.1136/thx.2003.014258 PMC1763796

[10] Tingpej P, Elkins M, Rose B, Hu H, Moriarty C, Manos J, et al Clinical profile of adult cystic fibrosis patients with frequent epidemic clones of *Pseudomonas aeruginosa*. Respirology 2010 8;15(6):923–9. 10.1111/j.1440-1843.2010.01792.x 20573059

[11] Salunkhe P, Smart CH, Morgan JA, Panagea S, Walshaw MJ, Hart CA, et al A cystic fibrosis epidemic strain of *Pseudomonas aeruginosa* displays enhanced virulence and antimicrobial resistance. J Bacteriol 2005 7;187(14):4908–20. 15995206 10.1128/JB.187.14.4908-4920.2005 PMC1169510

[12] Ashish A, Shaw M, Winstanley C, Ledson MJ, Walshaw MJ. Increasing resistance of the Liverpool Epidemic Strain (LES) of Pseudomonas aeruginosa (Psa) to antibiotics in cystic fibrosis (CF)—a cause for concern? J Cyst Fibros 2012 5;11(3):173–9. 10.1016/j.jcf.2011.11.004 22146482

[13] The Cystic Fibrosis Trust. Available: http://wwwcysticfibrosis.org.uk/about-cf/living-with-cystic-fibrosis/cross-infection. Accessed 2014.

[14] Dutch Institute for Healthcare Improvement. CBO Guideline Diagnosis and Treatment Cystic Fibrosis (Dutch). 2007.

[15] Dutch Cystic Fibrosis Foundation website. Available: http://www.ncfs.nl. Accessed 2013.

[16] Brimicombe RW, Dijkshoorn L, van der Reijden TJ, Kardoes I, Pitt TL, van den Broek PJ, et al Transmission of *Pseudomonas aeruginosa* in children with cystic fibrosis attending summer camps in The Netherlands. J Cyst Fibros 2008 1;7(1):30–6. 17532271 10.1016/j.jcf.2007.04.002

[17] van Mansfeld R, Willems R, Brimicombe R, Heijerman H, van Berkhout FT, Wolfs T, et al *Pseudomonas aeruginosa* genotype prevalence in Dutch cystic fibrosis patients and age dependency of colonization by various P. aeruginosa sequence types. J Clin Microbiol 2009 12;47(12):4096–101. 10.1128/JCM.01462-09 19828746 PMC2786644

[18] van Mansfeld R, Jongerden I, Bootsma M, Buiting A, Bonten M, Willems R. The population genetics of *Pseudomonas aeruginosa* isolates from different patient populations exhibits high-level host specificity. PLoS One 2010;5(10):e13482 10.1371/journal.pone.0013482 20976062 PMC2957436

[19] de Vrankrijker AM, Brimicombe RW, Wolfs TF, Heijerman HG, van MR, van Berkhout FT, et al Clinical impact of a highly prevalent *Pseudomonas aeruginosa* clone in Dutch cystic fibrosis patients. Clin Microbiol Infect 2011 3;17(3):382–5. 10.1111/j.1469-0691.2010.03295.x 20807225

[20] Jones AM, Dodd ME, Govan JR, Doherty CJ, Smith CM, Isalska BJ, et al Prospective surveillance for *Pseudomonas aeruginosa* cross-infection at a cystic fibrosis center. Am J Respir Crit Care Med 2005 2 1;171(3):257–60. 15542795 10.1164/rccm.200404-513OC

[21] Griffiths AL, Wurzel DF, Robinson PJ, Carzino R, Massie J. Australian epidemic strain pseudomonas (AES-1) declines further in a cohort segregated cystic fibrosis clinic. J Cyst Fibros 2012 1;11(1):49–52. 10.1016/j.jcf.2011.08.005 21907639

[22] Griffiths AL, Jamsen K, Carlin JB, Grimwood K, Carzino R, Robinson PJ, et al Effects of segregation on an epidemic *Pseudomonas aeruginosa* strain in a cystic fibrosis clinic. Am J Respir Crit Care Med 2005 5 1;171(9):1020–5. 15709051 10.1164/rccm.200409-1194OC

[23] Ashish A, Shaw M, Winstanley C, Humphreys L, Walshaw MJ. Halting the spread of epidemic *Pseudomonas aeruginosa* in an adult cystic fibrosis centre: a prospective cohort study. JRSM Short Rep 2013 1;4(1):1 10.1258/shorts.2012.012018 23413403 PMC3572656

[24] Frederiksen B, Koch C, Hoiby N. Changing epidemiology of *Pseudomonas aeruginosa* infection in Danish cystic fibrosis patients (1974–1995). Pediatr Pulmonol 1999 9;28(3):159–66. 10495331 10.1002/(sici)1099-0496(199909)28:3<159::aid-ppul1>3.0.co;2-1

[25] Wiehlmann L, Cramer N, Ulrich J, Hedtfeld S, Weissbrodt H, Tummler B. Effective prevention of *Pseudomonas aeruginosa* cross-infection at a cystic fibrosis centre—results of a 10-year prospective study. Int J Med Microbiol 2012 3;302(2):69–77. 10.1016/j.ijmm.2011.11.001 22196973

[26] Taccetti G, Bianchini E, Cariani L, Buzzetti R, Costantini D, Trevisan F, et al Early antibiotic treatment for Pseudomonas aeruginosa eradication in patients with cystic fibrosis: a randomised multicentre study comparing two different protocols. Thorax 2012 10;67(10):853–9. 22379071 10.1136/thoraxjnl-2011-200832

[27] Quanjer PH, Tammeling GJ, Cotes JE, Pedersen OF, Peslin R, Yernault JC. Lung volumes and forced ventilatory flows. Report Working Party Standardization of Lung Function Tests, European Community for Steel and Coal. Official Statement of the European Respiratory Society. Eur Respir J Suppl 1993 3;16:5–40. 8499054

[28] Koopman M, Zanen P, Kruitwagen CL, van der Ent CK, Arets HG. Reference values for paediatric pulmonary function testing: The Utrecht dataset. Respir Med 2011 1;105(1):15–23. 10.1016/j.rmed.2010.07.020 20889322

[29] Lee TW, Brownlee KG, Conway SP, Denton M, Littlewood JM. Evaluation of a new definition for chronic Pseudomonas aeruginosa infection in cystic fibrosis patients. J Cyst Fibros 2003 3;2(1):29–34. 15463843 10.1016/S1569-1993(02)00141-8

[30] EUCAST clinical breakpoints. Available: http://www.eucast.org/clinical_breakpoints/. Accessed 2013.

[31] Curran B, Jonas D, Grundmann H, Pitt T, Dowson CG. Development of a multilocus sequence typing scheme for the opportunistic pathogen *Pseudomonas aeruginosa*. J Clin Microbiol 2004 12;42(12):5644–9. 15583294 10.1128/JCM.42.12.5644-5649.2004 PMC535286

[32] Jolley KA, Maiden MC. BIGSdb: Scalable analysis of bacterial genome variation at the population level. BMC Bioinformatics 2010;11:595 10.1186/1471-2105-11-595 21143983 PMC3004885

[33] Francisco AP, Bugalho M, Ramirez M, Carrico JA. Global optimal eBURST analysis of multilocus typing data using a graphic matroid approach. BMC Bioinformatics 2009;10:152 10.1186/1471-2105-10-152 19450271 PMC2705362

[34] Francisco AP, Vaz C, Monteiro PT, Melo-Cristino J, Ramirez M, Carrico JA. PHYLOViZ: phylogenetic inference and data visualization for sequence based typing methods. BMC Bioinformatics 2012;13:87 10.1186/1471-2105-13-87 22568821 PMC3403920

[35] Schoenfeld DA. Sample-size formula for the proportional-hazards regression model. Biometrics 1983 6;39(2):499–503. 6354290

[36] Liu G, Liang KY. Sample size calculations for studies with correlated observations. Biometrics 1997 9;53(3):937–47. 9290224

[37] CFBD. Available: http://www.cfbeachdance.nl/. Accessed 2014.

[38] Hansen SK, Rau MH, Johansen HK, Ciofu O, Jelsbak L, Yang L, et al Evolution and diversification of *Pseudomonas aeruginosa* in the paranasal sinuses of cystic fibrosis children have implications for chronic lung infection. ISME J 2012 1;6(1):31–45. 10.1038/ismej.2011.83 21716309 PMC3246239

[39] Johansen HK, Aanaes K, Pressler T, Nielsen KG, Fisker J, Skov M, et al Colonisation and infection of the paranasal sinuses in cystic fibrosis patients is accompanied by a reduced PMN response. J Cyst Fibros 2012 12;11(6):525–31. 10.1016/j.jcf.2012.04.011 22595452

[40] Aanaes K, Johansen HK, Skov M, Buchvald FF, Hjuler T, Pressler T, et al Clinical effects of sinus surgery and adjuvant therapy in cystic fibrosis patients—can chronic lung infections be postponed? Rhinology 2013 9;51(3):222–30. 10.4193/Rhino12.207 23943728

[41] Cutting GR. Cystic fibrosis genetics: from molecular understanding to clinical application. Nat Rev Genet 2015 1;16(1):45–56. 10.1038/nrg3849 25404111 PMC4364438

[42] Green DM, Collaco JM, McDougal KE, Naughton KM, Blackman SM, Cutting GR. Heritability of respiratory infection with Pseudomonas aeruginosa in cystic fibrosis. J Pediatr 2012 8;161(2):290–5. 10.1016/j.jpeds.2012.01.042 22364820 PMC3682831

[43] Emond MJ, Louie T, Emerson J, Zhao W, Mathias RA, Knowles MR, et al Exome sequencing of extreme phenotypes identifies DCTN4 as a modifier of chronic Pseudomonas aeruginosa infection in cystic fibrosis. Nat Genet 2012 8;44(8):886–9. 10.1038/ng.2344 22772370 PMC3702264

