# Supplementary figures and images for: The Effect of Strict Segregation on Pseudomonas aeruginosa in Cystic Fibrosis Patients
Source: PLoS One. 2016 Jun 9;11(6):e0157189. doi: 10.1371/journal.pone.0157189 (PMC4900627; doi:10.1371/journal.pone.0157189)

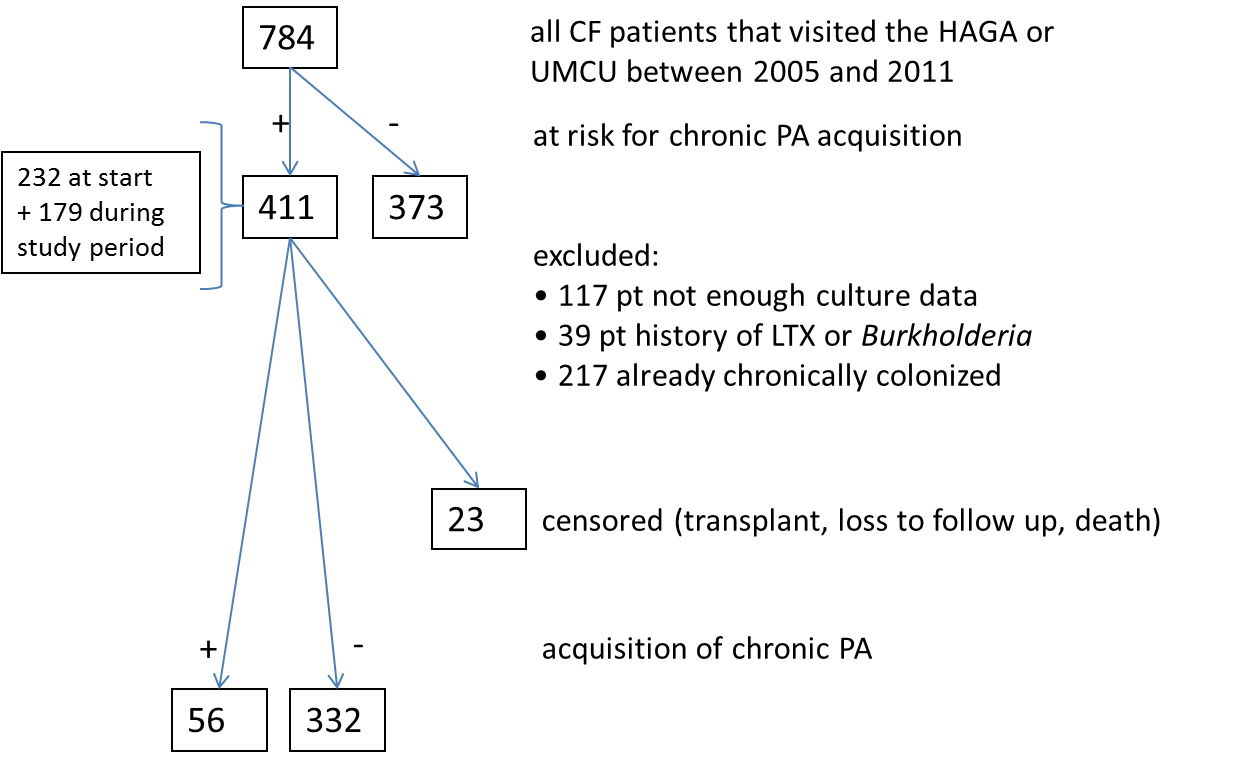

Supplement: S1 Fig — (TIF) [file pone.0157189.s001.tif]

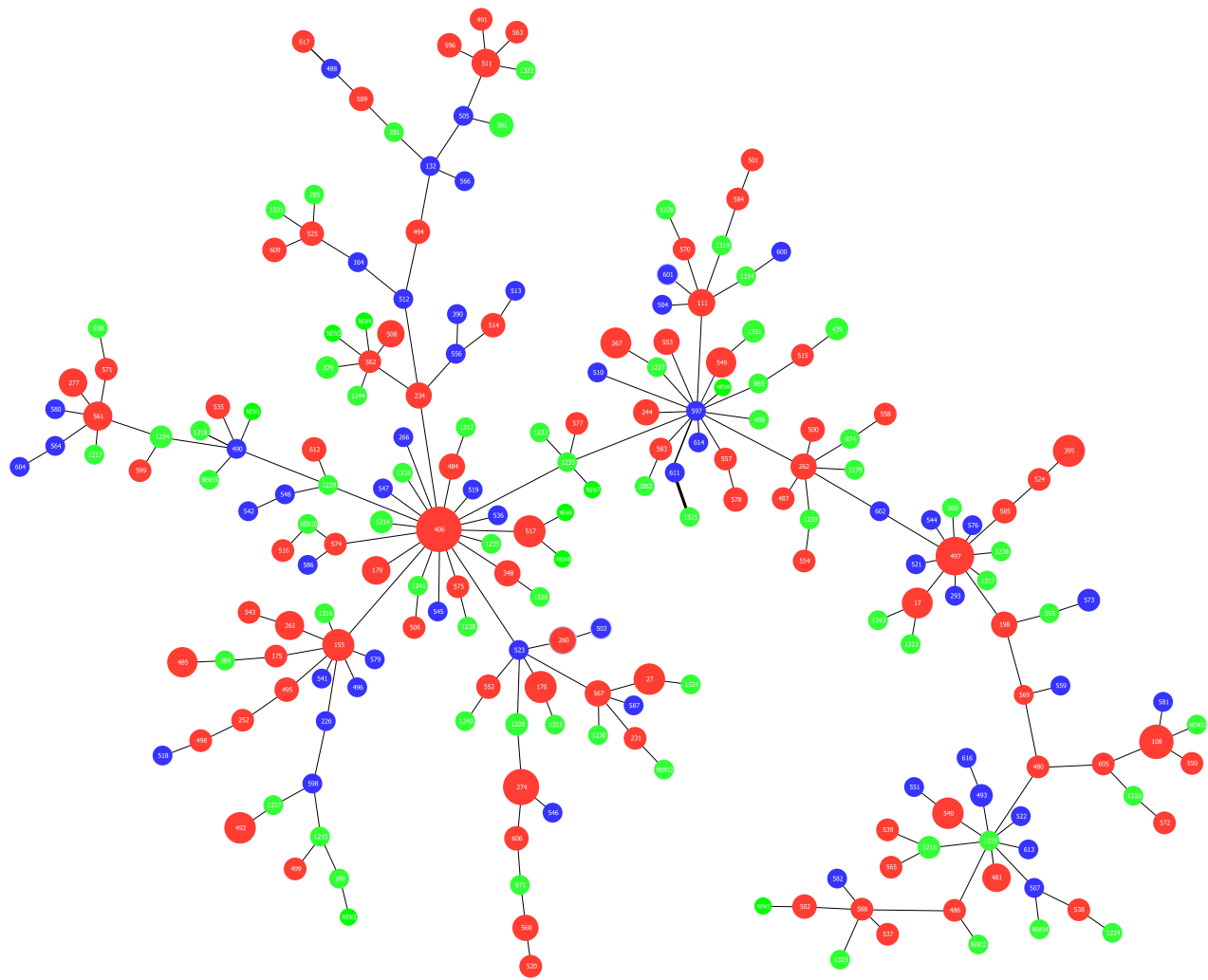

Supplement: S2 Fig — Genetic linkage was done using goeBURST distances[33] Each circle represents a sequence type, the size of the circle represents the number of isolates (non-linear). Color of the circle indicates year of origin; blue: 2007 only, green: 2011 only, red: isolated in both 2007 and 2011. Edges connect genetically linked STs. (PDF) [file pone.0157189.s002.pdf]

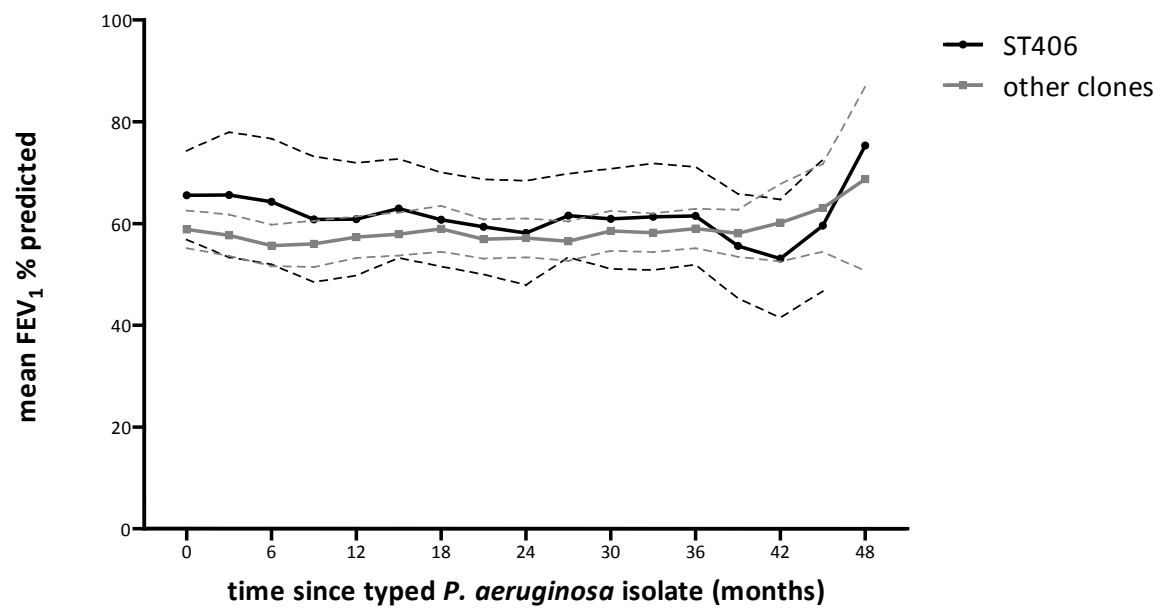

Supplement: S3 Fig — Dotted lines represent 95% CI’s. Longitudinal lung function measurements (mean FEV1 as percentage of predicted) for patients with ST406 and patients with other clones. There was no significant difference in lung function for patients with or without ST406 (estimate -0·43, 95% CI -7·84–6·98). Adding an interaction term between ST406 and time (slope analysis, not shown in figure) did not significantly improve the model (p = 0·35) indicating no difference in decline. (PDF) [file pone.0157189.s003.pdf]
