# Supplementary material for: The Effect of Strict Segregation on Pseudomonas aeruginosa in Cystic Fibrosis Patients
Source: PLoS One. 2016 Jun 9;11(6):e0157189. doi: 10.1371/journal.pone.0157189 (PMC4900627; doi:10.1371/journal.pone.0157189)
Supplement: S1 Table — PA = P. aeruginosa, LTX = lung transplantation, * at risk are all patients without chronic PA infection. (DOCX) [file pone.0157189.s004.docx]

**Supplementary Table S1** :**CF patients at risk for chronic colonization with *P. aeruginosa* between 2005-2011**

| Year | At risk for chronic infection PA on 1^st^of January(n)* | New patients at risk for chronic infection with PA, included during study period (n) | Acquisitions of chronic infection with PA(n) | Lost to follow-up  (n) |
| --- | --- | --- | --- | --- |
| 2005 | 232 | 57 | 11 | 0 |
| 2006 | 278 | 26 | 17 | 1 |
| 2007 | 286 | 20 | 13 | 2  (LTX, n=1) |
| 2008 | 291 | 18 | 5 | 4  (LTX, n=1, death n=1) |
| 2009 | 300 | 25 | 5 | 6  (death, n=2) |
| 2010 | 314 | 21 | 3 | 10 |
| 2011 | 322 | 12 | 2 | 0 |

PA=*P. aeruginosa*, LTX= lung transplantation, * at risk are all patients without chronic PA infection
