# Supplementary material for: The Effect of Strict Segregation on Pseudomonas aeruginosa in Cystic Fibrosis Patients
Source: PLoS One. 2016 Jun 9;11(6):e0157189. doi: 10.1371/journal.pone.0157189 (PMC4900627; doi:10.1371/journal.pone.0157189)
Supplement: S2 Table — (DOCX) [file pone.0157189.s005.docx]

**S2 Table. Estimates of FEV_1_ percent of predicted based on mixed model analysis**

|  | **unadjusted** | | **adjusted** | |
| --- | --- | --- | --- | --- |
| **variable** | **estimate** | **95% CI** | **estimate** | **95% CI** |
| intercept | 59·17 | [55·75, 62·58] | 85·32 | [77·45, 93·20] |
| ST406 | 3·60 | [-4·35, 11·54] | -0·43 | [-7·84, 6·98] |
| slope (ST406*time) | -0·59 | [-1·80, 0·61] | -0·57 | [-1·77, 0·63] |
| using inhaled antibiotics |  |  | -10·05 | [-15·94, -4·16] |
| time (in years) | -1·19 | [-1·71, -0·67] | -0·45 | [-1·02, 0·12] |
| age (in years) |  |  | -0·75 | [-1·00, -0·51] |
